# Effect of a Multi-Level Education Intervention Model on Knowledge and Attitudes of Accidental Injuries in Rural Children in Zunyi, Southwest China

**DOI:** 10.3390/ijerph120403903

**Published:** 2015-04-08

**Authors:** Bo-Ling Cao, Xiu-Quan Shi, Yong-Hong Qi, Ya Hui, Hua-Jun Yang, Shang-Peng Shi, Li-Rong Luo, Hong Zhang, Xin Wang, Ying-Ping Yang

**Affiliations:** 1 Department of Epidemiology and Health Statistics, School of Public Health, Zunyi Medical University, Zunyi 563099, China; E-Mails: shuannimeng@163.com (B.-L.C.); qyhwanan@yeah.net (Y.-H.Q.); vinald1988@126.com (Y.H.); yanghuajun0201@163.com (H.-J.Y.); shangpengss@163.com (S.-P.S.); 2 Affiliated Hospital of Zunyi Medical University, Zunyi 563099, China; E-Mails: zylirongluo@163.com (L.-R.L.); zhanghong19861208@163.com (H.Z.); xwang@zmc.edu.cn (X.W.); 3 School of Management, Zunyi Medical University, Zunyi 563099, China; E-Mail: ypyang57@126.com

**Keywords:** rural children, accidental injury, intervention, knowledge and attitude

## Abstract

*Objective*: To explore the effect of a school-family-individual (SFI) multi-level education intervention model on knowledge and attitudes about accidental injuries among school-aged children to improve injury prevention strategies and reduce the incidence of pediatric injuries. *Methods*: The random sample of rural school-aged children were recruited by using a multistage, stratified, cluster sampling method in Zunyi, Southwest China from 2012 to 2014, and 2342 children were randomly divided into intervention and control groups. Then children answered a baseline survey to collect knowledge and attitude scores (KAS) of accidental injuries. In the intervention group, children, their parents/guardians and the school received a SFI multi-level education intervention, which included a children’s injury-prevention poster at schools, an open letter about security instruction for parents/guardians and multiple-media health education (Microsoft PowerPoint lectures, videos, handbooks, *etc.*) to children. Children in the control group were given only handbook education. After 16 months, children answered a follow-up survey to collect data on accidental injury types and accidental injury-related KAS for comparing the intervention and control groups and baseline and follow-up data. *Results*: The distribution of gender was not significantly different while age was different between the baseline and follow-up survey. At baseline, the mean KAS was lower for the intervention than control group (15.37 ± 3.40 and 18.35 ± 5.01; *p* < 0.001). At follow-up, the mean KAS was higher for the intervention than control group (21.16 ± 3.05 and 20.02 ± 3.40; *p* < 0.001). The increase in KAS in the intervention and control groups was significant (*p* < 0.001; KAS: 5.79 *vs.* 1.67) and suggested that children’s injury-related KAS improved in the intervention group. Moreover, the KAS between the groups differed for most subtypes of incidental injuries (based on International Classification of Diseases 10, ICD-10) (*p* < 0.05). Before intervention, 350 children had reported their accident injury episodes, while after intervention 237 children had reported their accidental injury episodes in the follow-up survey. *Conclusions*: SFI multi-level education intervention could significantly increase KAS for accidental injuries, which should improve children’s prevention-related knowledge and attitudes about such injuries. It should help children change their risk behaviors and reduce the incidence of accidental injuries. Our results highlight a new intervention model of injury prevention among school-aged children.

## 1. Introduction

According to the World Health Organization (WHO), accidental injury is one of the major causes of death around the world, and accidental injury is the main reason for death in children <15 years old [1]. In some developing countries, 80% of the total mortality in children is due to accidental deaths [2]. As the most populous developing country, China is experiencing an emerging public health problem of injuries, which cause almost 700,000 to 750,000 deaths each year, with a death rate of 65.2/100,000 inhabitants [3]. The annual number of deaths from injuries in China is estimated to reach 1,400,000 by 2010 and 2,500,000 by 2050 [4]. 

Moreover, accidental injury is the main reason for children’s disability. Every year, millions of children around the world are hurt and even disabled because of accidental injuries [2,5]. Meanwhile, because of accidental injury, children may have physical and mental developmental disorders such as resentment, blame, and feelings of guilt [6]. Consequently, specific interventions should focus on children’s accidental injuries [7,8].

Children in rural areas of China are more vulnerable to injury than those in urban areas and might have different injury patterns as compared with urban children [9]. As a typical representative rural area in a western mountainous area in China, injury-related research in Guizhou Province was very weak [10]. According to the monitoring data offered by the Guizhou Center of Disease Control and Prevention, at the end of 2007, the accidental injury rate was 24.1% among children ≤15 years old; however, related research on injury intervention is scarce [11]. At present, research into children’s accidental injury has focused on some injury-related intervention strategies, with few quasi-experimental or experimental research intervention studies with well-designed interventions and surveys [12]. Health education is considered a low-cost and highly effective intervention [13]. Some studies found that children’s safety consciousness is improved by health education [14], but whether some other intervention models can effectively prevent children’s injuries is still unclear. Recent evidence suggests that the impact of safety education for children on unintentional injury rates is needed [15].

Most domestic intervention research [16] used only student education, multiple factors; health education for injury intervention should be extended to many players in children’s lives, especially people they are in close contact with, including their parents, teachers and classmates.

In this study, we aimed to explore the effect of a multi-level education injury intervention model on knowledge and attitudes about accidental injury in rural children in a mountainous region of Southwest China. We wanted to test whether children’s knowledge and attitudes about accidental injuries can be effectively improved by a multi-level education intervention model, school-family-individual (SFI) education.

## 2. Methods

### 2.1. Research Subjects

This study adopted multistage, stratified, cluster, and probability proportional to size cluster sampling for the investigation. In the first stage, two primary (the age is between 8–14) and three middle schools (the age is between 10–16) were randomly selected from a random sample of Meitan and Zheng’an counties in Zunyi (a rural mountainous area in Southwest China); in the second stage, Grade 4 to 6 students were randomly selected from the primary school and Grade 7 to 8 students from the middle school. All participating subjects gave their informed consent to participate in the study.

### 2.2. Sample Size Estimation

In our survey, we evaluated the sample size by formula [17]:

N = n_t_ × number of strata = (Z_α_ + Z_β_)^2^ × (σ_I_^2^ + σ_C_^2^)/δ^2^ × 120% × number of strata

In the formula: 

n_t_ is the theoretic sample size of one stratum; α is the significance level, let α = 0.05, Z_0.05_ = 1.96; β is the type II error, 1-β is the power of a significance test, let power = 0.9, β = 0.1, so Z_0.1_ = 1.282; σ_I_^2^ and σ_C_^2^ are the standard deviation (SD) of the intervention group and control group; δ (delta) is the difference of population curative effect between the intervention group and the control group; Let δ = σ_I_ = σ_C_.

Considered the missing value or non-response, we added 20% more to sample, so we multiply by 120% the sample size estimation. Considering there might be a potential difference of injury rate between boys and girls; we prepared to divide the children into two strata. Moreover, as injuries include several subtypes, such as traffic accidents, falls, burns, the number of strata should be substantially increased. The final estimated sample size was 832, and our actual sample size was enough to ensure acceptable statistical power.

### 2.3. Investigation Method

Data for all subjects of the first survey, which investigated the related situations, knowledge and attitudes of the accidental injury that occurred in the previous year, were used as baseline data. Data for all subjects of the second survey, conducted 16 months later (our intervention time lasted from November 2012 to March 2014), which investigated the related situations, knowledge and attitudes of the accidental injury that occurred after the first survey, were used as follow-up data. 

Our study adopted a self-designed injury questionnaire, which was revised by the preliminary experiment. The questionnaire consisted of four parts: basic information, family situation and basic situation and related knowledge of the accidental injury events. Students independently completed the questionnaire with instructions from trained investigators in one session. The total knowledge and attitudes score (KAS) for awareness of the situation was 25 points, with 1 for a correct answer, and 0 for an incorrect answer on the survey.

### 2.4. Definition and Classification Criteria of Injuries

Injuries were defined as: (1) a diagnosis in a hospital; (2) emergency treatment or care by parents, teachers, classmates or partner; and (3) asking to leave school for more than half a day because of injury (stop activity, rest) to determine an injury that met any of the above conditions [18]. The baseline survey collected data on the injury condition and injury-related knowledge and attitudes in the previous 12 months and the follow-up survey collected these data for the injury that occurred since the baseline survey. Accidental injuries were classified [19] according the ICD-10, which includes drowning, falls, burns, traffic accidents, poisoning and asphyxia and other related injury accidents (e.g., sharp object injury, firearm wounding, machine injury, natural and environmental damage, crushing injury).

### 2.5. Intervention

The intervention comprised a 16-month multi-level education intervention in students: (1) an open letter distributed to parents/other guardians discussed precautions, helping parents find family risk factors for the susceptible accidental injuries for children and prevention and emergency treatment of accidental injuries; (2) a “handbook” and poster to prevent school-age children accidental injuries were distributed to students to strengthen safety awareness; (3) lectures and video education about accidental injuries were provided to students and teachers to help avoid injury-related risk behaviors. Moreover, teachers were trained in the prevention and emergency treatment of minor accidental injuries in children. 

### 2.6. Data Collection and Quality Control

Data were collected at each school by using a standard questionnaire. Professional interviewers entered each classroom, explained the purpose of the survey, and obtained informed consent from the students who participated in this survey. The principal researchers visited the field to supervise the survey activities. The principal researchers and all interviewers reviewed the completed questionnaires for accuracy and completeness during the surveys.

### 2.7. Ethics Statement

Written informed consent was obtained from each student and parents or legal guardian(s). The research protocol and questionnaires were approved by the 5targeted schools and the Institutional Review Board of Zunyi Medical University (No. ZYMEC 2013-001).

### 2.8. Statistical Analysis

A database was constructed by use of EpiData 3.1 (http://www.epidata.dk). Data were double-input to reduce errors. SPSS v18.0 (SPSS Inc., Chicago, IL, USA) was used for statistical analyses. Data are described with numbers or mean ± SD; Student *t* test and chi-square test were used to compare differences in control and intervention groups. *p* < 0.05 was considered statistically significant.

## 3. Results

### 3.1. The General Characters of Students

For the baseline survey, we issued 2351 questionnaires and received 2342 complete questionnaires (effective response rate 99.6%). Further follow-up survey, we issued 1506 questionnaires and received 1502 complete questionnaires (effective response rate 99.7%). In total, 2342 students participated in the baseline survey (50.13% boys); 1502 students participated in the follow-up survey (51.53% boys) (Table 1). The intervention and control groups did not differ in sex at baseline (*p* = 0.693) or follow-up (*p* = 0.631) (Table 1). The proportion of primary and junior middle school students differed at baseline (*p* < 0.001) but not follow-up (*p* = 0.095). The mean age differed between groups for the two survey stages (*p* < 0.001).

### 3.2. Effect of Family Environment on KAS

Children in a one-child family, with a main guardian and education level of guardians all affected KAS of accidental injury (Table 2). Moreover, KAS differed between control and intervention groups.

### 3.3. Effect of the Intervention on KAS and Injury Incidence

The KAS was lower for the intervention and control groups at baseline (15.37 ± 3.40 and 18.35 ± 5.01) than that in follow-up (21.16 ± 3.05 and 20.02 ± 3.40). The KAS differed between the control and intervention groups at baseline and follow-up (*p* < 0.001), and differed from baseline to follow-up for the intervention and control groups (*p* < 0.001) (Table 3).

At baseline, the mean KAS for accidental injury, including traffic, falls, burns, animal bites, electric shock, poisoning, and asphyxia but not falls or drowning, statistically significantly differed between intervention and control groups. At follow-up, the mean KAS for accidental injury, including traffic, falls, burns, and asphyxia but not animal bites, electric shock, poisoning or drowning, statistically significantly differed. 

**Table 1:** Basic characteristics of students for baseline and follow-up surveys of knowledge and attitudes about accidental injury.

Demographic	Baseline Survey		Follow-Up Survey	
	Intervention Group (*n* = 879)	Control Group (*n* = 1463)	Intervention Group (*n* = 841)	Control Group (*n* = 661)
**Gender**				
Male	436(49.6)	738(50.4)	438(52.1)	336(50.8)
Female	443(50.4)	725(49.6)	403(47.9)	325(49.2)
*Chi-*square values	0.16		0.23	
*p*-value	0.693		0.631	
**Grade**				
Primary school	554(63.0)	521(35.6)	517(61.5)	434(65.7)
Middle school	325(37.0)	942(64.4)	324(38.5)	227(34.3)
*Chi-square values*	166.19		2.79	
*p-value*	<0.001		0.095	
**Age**, mean ± SD	11.91 ± 1.45	12.61 ± 1.54	13.33 ± 1.40	12.59 ± 1.72
*Z-value*	388.09		292.77	
*p-value*	<0.001		<0.001	

Data are number (%) of students unless indicated.

**Table 2:** Knowledge and attitude scores (KAS) for children from different family environments in the intervention and control groups at baseline and follow-up.

Factors	Intervention Group					Control Group					*t*_3_
	Baseline Survey		Follow-up Survey		*t*_1_	Baseline Survey		Follow-up Survey		*t*_2_	
	No.	Scores	No.	Scores		No.	Scores	No.	Scores		
**One-child family**											
Yes	152	15.19 ± 3.58	126	21.03 ± 2.82	14.88 **	208	18.84 ± 4.14	122	19.75 ± 3.94	1.97 *	5.18 ^##^
No	702	15.49 ± 3.22	689	21.24 ± 3.08	33.94 **	1205	18.42 ± 4.97	525	20.15 ± 3.16	7.36 **	10.11 ^##^
**Main guardian**											
Parents together	353	15.64 ± 3.37	383	21.38 ± 2.90	24.82 **	571	18.96 ± 4.55	318	20.46 ± 3.02	5.26 **	7.98 ^##^
Father only	36	14.83 ± 3.28	32	21.62 ± 2.65	10.16 **	115	18.25 ± 4.35	44	19.52 ± 3.74	1.71	3.33 ^##^
Mother only	163	15.03 ± 3.83	164	20.88 ± 3.33	14.76 **	235	18.18 ± 5.28	87	20.10 ± 3.13	3.19 *	3.70 ^##^
Other relatives	270	15.60 ± 2.61	217	21.13 ± 2.71	22.79 **	505	18.14 ± 5.05	186	19.58 ± 3.67	3.55 **	7.51 ^##^
**Guardian education**											
>High school	137	15.56 ± 3.60	121	20.80 ± 3.35	12.06 **	226	18.66 ± 4.64	104	20.05 ± 3.89	2.64 *	3.54 ^##^
Middle school	406	15.55 ± 3.21	394	21.53 ± 2.83	27.88 **	592	18.58 ± 4.81	308	20.46 ± 2.93	6.29 **	8.45 ^##^
Primary school	196	14.92 ± 3.49	224	21.08 ± 3.0	19.32 **	428	18.38 ± 4.96	160	19.62 ± 3.23	2.94 *	6.23 ^##^
Illiterate	86	15.52 ± 3.01	78	20.62 ± 3.23	10.44 **	126	18.48 ± 4.64	55	20.15 ± 3.53	2.39 *	3.05 ^##^

Data are number or mean ± SD. Note: *t*_1_ is baseline *vs.* follow-up in intervention group; *t*_2_ is baseline *vs.* follow-up in control group; *t*_3_ is KAS for control *vs.* intervention group, KAS is change in score before and after intervention/scores of follow-up minus baseline; * *p* < 0.05, ** *p* < 0.001 comparing baseline *vs.* follow-up; ^#^ KAS for control *vs.* intervention group, *p* < 0.05, ^##^ *p* < 0.001.

**Table 3:** Mean KAS for accidental injury types before and after intervention.

Injury Type	Subtotal Scores	Baseline Survey				Follow-up Survey				*t*_3_
		Intervention Group	Control Group	*t*_1_	*p*	Intervention Group	Control Group	*t*_2_	*p*	
Traffic	5	2.09 ± 0.86	3.53 ± 1.37	28.12	<0.001	4.47 ± 0.82	4.26 ± 1.03	4.42	<0.001	65.87 ^##^
Falls	2	1.29 ± 0.69	1.33 ± 0.73	1.33	0.184	1.59 ± 0.58	1.09 ± 0.68	15.38	<0.001	36.86 ^##^
Burns	6	4.26 ± 1.24	4.55 ± 1.50	4.86	<0.001	5.41 ± 0.94	5.18 ± 1.10	4.39	<0.001	14.64 ^##^
Animal bites	2	1.57 ± 0.65	1.44 ± 0.73	4.40	<0.001	1.52 ± 0.57	1.12 ± 0.56	1.06	0.292	18.56 ^##^
Electric shock	3	0.94 ± 0.24	1.65 ± 0.51	39.08	<0.001	2.39 ± 0.74	2.40 ± 0.74	0.45	0.650	75.75 ^##^
Poisoning	3	1.72 ± 0.54	2.48 ± 0.82	24.67	<0.001	2.65 ± 0.58	2.66 ± 0.65	0.03	0.973	66.40 ^##^
Asphyxia	2	1.76 ± 0.52	1.66 ± 0.61	4.13	<0.001	1.80 ± 0.46	1.66 ± 0.60	5.12	<0.001	4.59 ^##^
Drowning	2	1.75 ± 0.54	1.70 ± 0.60	1.80	0.730	1.70 ± 0.49	1.65 ± 0.53	1.86	0.063	0.60
Total	25	15.37 ± 3.40	18.35 ± 5.01	15.61	<0.001	21.16 ± 3.05	20.02 ± 3.40	6.86	<0.001	11.30 ^##^

*t*_1_ is intervention *vs.* control group at baseline; *t*_2_ is intervention *vs.* control group at follow-up; *t*_3_ is KAS for intervention *vs.* control group, KAS as in Table 2; *^##^* KAS for intervention *vs.* control group, *p* < 0.001.

Except for drowning-related KAS, the increased KAS in the intervention and control groups significantly differed between before and after the intervention. In summary, the increased KAS scores (KAS: 5.79 *vs*. 1.67) in the intervention and control groups statistically significantly differed (*p* < 0.001), which suggested that injury-related KAS improved more in the intervention than control group (Table 3).

Before intervention, 350 children had reported their accident injury episodes while after intervention 237 children had reported their accident injury episodes in the follow-up survey. At baseline, the accidental injury incidence was 15.02% in the intervention group and 14.90% in the control group. At follow-up, the annual rates were 11.04% and 12.84%, respectively. After the intervention, the injury incidence decreased with increasing KAS but not significantly different between two groups (*p* > 0.05).

## 4. Discussion

Previous epidemiological surveys suggested that children in the primary and middle school stages have the strongest vitality and the least physical illness in life, but it is a vulnerable age for unintentional injury [20,21]. Our previous studies showed that the annual incidence of unintentional injury was 16.7% in Zunyi in 2012 [22]. In this study, we aimed to explore the effect of a SFI multi-level education intervention model on KAS for accidental injuries among school-aged children to improve injury prevention strategies and reduce the incidence of pediatric injuries. We found that KAS for accidental injuries were higher among children who received a SFI multi-level education intervention for 16 months than those in control group.

Studies showed that most of accidental injuries in primary and middle school students can be avoided by a prevention strategy, especially minor accidental injuries [23,24]. Moreover, the injury causes were generally associated with students’ activities such as playing and sports, so most of unintentional injuries could be prevented by strengthening safety health education, taking related intervention measures and creating a safe living and learning environment [25]. In 2010, Zhang and colleagues performed health education activities to improve students’ knowledge of injury awareness and self-precaution consciousness in some primary and middle schools in Hongqiao District, Tianjin City [26].

The results of our survey suggested that children in different family environments had increased understanding of unintentional injuries after the intervention, with a role for the main guardian in this. Although the mean KAS was increased after the multi-level education intervention, KAS for children with parents as main guardians were lower for unintentional injuries than other kinds of main guardians. The effect of the SFI multi-level education intervention may have increased the children’s knowledge about accidental injuries, especially if guardians were parents. Children with guardians with increased education had better mastery of accidental injury, which is consistent with a previous study [27]. Low literacy of parents may affect children’s health and safety education, with poor understanding of the causes of children’s accidental injuries. 

The mean KAS was higher in the control than intervention group at baseline, but after 16 months of the SFI intervention, the change in KAS was higher for the intervention than control group (KAS: 5.79 *vs*. 1.67, last row in Table 3). Therefore, the SFI multi-level education intervention, including three levels of health education, may have improved the understanding of accidental injuries among children. 

Studies of children’s injury interventions in China showed that children may improve their knowledge of injury prevention and self-precaution consciousness by taking multi-level education intervention measures (mainly focusing on health education and safety promotion). This should effectively reduce the injury incidence among primary and middle school students [28,29,30]. In New Zealand, a cluster-randomized controlled trial of households found injuries specific to the home-modification intervention were cut by 39% per year exposed (0.61, 0.41–0.91). Their findings suggest that low-cost home modifications intervention can be a means to reduce injury especially falls [31]. Knowledge of children’s safety in crossing the road improved. Morrongiello and colleagues [32] performed the “Practice what you preach” project, which displayed behaviors that students easily cause injuries on the playground in the form of posters; students’ understandings of the accident injuries that occur in the playground were improved and risk behaviors were reduced. Furthermore, the United Kingdom carried out the “Risk Watch” project [14], which has significantly improved students’ safety knowledge, safety skills and self-reported attitudes. In this study, we found that children improved their understandings of accidental injury and acquired better KAS after our SFI multi-level education intervention, which agrees with other study findings in China [33,34,35].

Our study has some limitations. First, injury-related information, knowledge and attitudes were mainly from self-reports, and some children possibly forgot the exact situation if they were injured in the last year, and recall bias cannot be avoided. Moreover, as younger children have less study pressure, they are better to insist on a 16-month intervention and follow up, thus, children we selected in the intervention group were a little younger than the control group. Though the compliance was enhanced, other bias especially selection bias also cannot be avoided. Second, the KAS of accidental injury significantly increased after our multi-level health education intervention; however, it did not equal to their behaviors (practices) should be changed in some children. Just as some doctors still smoke though they know the harmfulness of smoking. Last, although we used several means to promote injury prevention for 16 months, this is still a short intervention time. In this period, children’s KAS increased and injury incidence decreased, although not significantly. Therefore, a 3-year intervention may be needed and so ensure the short-, medium- and long-term effect of the intervention measures by the time series method, which may provide more sufficient evidence for injury intervention effect [36,37].

Many countries emphasized injury research in the early 1980s and performed various types of injury monitoring; so a few countries have established a monitoring system [23]. However, many poor areas in China do not have an injury surveillance system for children. Our country should learn from other countries combined with our country-specific national conditions to improve monitoring [38]. 

Students’ injury problems should attract much attention from schools, education and health bureaus and even the whole society. Moreover, the school is a good environment to conduct health education interventions. The school should carry out health education and promotion for injury prevention in children. We recommend an SFI multi-level education intervention model to improve children’s knowledge and attitudes about accidental injuries and strengthen their risk precaution ability and basic skills to prevent the unintentional injury.

## 5. Conclusions

The SFI multi-level education intervention could significantly increase KASs for accidental injuries, which should improve children’s prevention-related knowledge and attitudes about such injuries. Our results highlight a new intervention model of injury prevention among school-aged children.
